# Sociodemographic and Built Environment Associates of Travel to School by Car among New Zealand Adolescents: Meta-Analysis

**DOI:** 10.3390/ijerph17239138

**Published:** 2020-12-07

**Authors:** Sandra Mandic, Erika Ikeda, Tom Stewart, Nicholas Garrett, Debbie Hopkins, Jennifer S. Mindell, El Shadan Tautolo, Melody Smith

**Affiliations:** 1 School of Sport and Recreation, Faculty of Health and Environmental Sciences, Auckland University of Technology, Private Bag 92006, Auckland 1142, New Zealand; 2 Active Living Laboratory, School of Physical Education, Sport and Exercise Sciences, University of Otago, P.O. Box 56, Dunedin 9054, New Zealand; 3 Centre for Sustainability, University of Otago, P.O. Box 56, Dunedin 9054, New Zealand; 4 MRC Epidemiology Unit, University of Cambridge, Box 285 Institute of Metabolic Science Cambridge Biomedical Campus, Cambridge CB2 0QQ, UK; erika.ikeda@mrc-epid.cam.ac.uk; 5 Human Potential Centre, Auckland University of Technology, Private Bag 92006, Auckland 1142, New Zealand; tom.stewart@aut.ac.nz; 6 Department of Biostatistics and Epidemiology, Faculty of Health and Environmental Sciences, Auckland University of Technology, Private Bag 92006, Auckland 1142, New Zealand; nick.garrett@aut.ac.nz; 7 Transport Study Unit, School of Geography and the Environment, University of Oxford, South Parks Road, Oxford OX1 3QY, UK; debbie.hopkins@ouce.ox.ac.uk; 8 Department of Epidemiology and Public Health, UCL (University College London), 1–19 Torrington Place, London WC1E 6BT, UK; j.mindell@ucl.ac.uk; 9 Pacific Health Research Centre, School of Public Health & Interdisciplinary Studies, Auckland University of Technology, Private Bag 92006, Auckland 1142, New Zealand; dan.tautolo@aut.ac.nz; 10 School of Nursing, Faculty of Medical and Health Sciences, The University of Auckland, Private Bag 92019, Auckland 1142, New Zealand; melody.smith@auckland.ac.nz

**Keywords:** transport, school, driving, built environment, adolescents, meta-analysis

## Abstract

Travelling to school by car diminishes opportunities for physical activity and contributes to traffic congestion and associated noise and air pollution. This meta-analysis examined sociodemographic characteristics and built environment associates of travelling to school by car compared to using active transport among New Zealand (NZ) adolescents. Four NZ studies (2163 adolescents) provided data on participants’ mode of travel to school, individual and school sociodemographic characteristics, distance to school and home-neighbourhood built-environment features. A one-step meta-analysis using individual participant data was performed in SAS. A final multivariable model was developed using stepwise logistic regression. Overall, 60.6% of participants travelled to school by car. When compared with active transport, travelling to school by car was positively associated with distance to school. Participants residing in neighbourhoods with high intersection density and attending medium deprivation schools were less likely to travel to school by car compared with their counterparts. Distance to school, school level deprivation and low home neighbourhood intersection density are associated with higher likelihood of car travel to school compared with active transport among NZ adolescents. Comprehensive interventions focusing on both social and built environment factors are needed to reduce car travel to school.

## 1. Introduction

The pervasiveness and resilience of private, motorised vehicles as the dominant mode of transportation has been well documented [1,2]. The system of automobility [1] has a complex relationship with the built environment, social norms and expectations, policy and regulation [3]. Dependence on motorised transport contributes to numerous environmental and health-related issues. Despite increasing numbers of electric vehicles, fossil fuels remain the main energy source for road-based transport [4]. Road-transport accounts for 73% of transport-related greenhouse gas emissions, producing approximately a quarter of direct carbon emissions from fuel combustion [5,6], with little change over time [7]. Motorised vehicles have replaced shared, public travel modes (e.g., buses, trams, trains) and active travel modes (e.g., walking, cycling) even for short distances. Worldwide, people are using less bodily “metabolic” energy, and more energy from fossil fuels and internal combustion engines for travel, including to school.

Private vehicle ownership and use dominate the transport system in Aotearoa New Zealand (NZ). With one of the highest rates of private vehicle ownership among the Organisation for Economic Co-operation and Development (OECD) countries [8], in 2016–2017, 82% of all trip legs (across all travel purposes) in NZ were made by private vehicles [9], including one-third of trips within “walkable” distance (less than 2 km) [9,10]. Thus, motorised transport for school travel is widespread. Since 1989/1990, the rates of car travel to school have increased (from 21% in 1989/1990 to 32% in 2010–2014) while the rates of active transport to school have declined [11]. In NZ, adolescents 16 years of age and older are eligible to start the learning-to-drive process [12].

The dominance of private vehicle transport in NZ has significant implications for health, as well as having environmental and economic costs. NZ is rated 14th highest of 168 countries worldwide for the prevalence of insufficient physical activity among adults [13], with the third highest adult obesity rate in OECD countries [14]. NZ’s children and adolescents have high levels of physical inactivity and sedentary behaviours [15], and approximately one-third are overweight or obese [16]. NZ also has among the highest rates of road traffic injury and death among OECD countries [17]. Transport produces 17% of NZ’s greenhouse gas emissions [18]. Road vehicle emissions in NZ increased by 82% between 1990 and 2016, and made up 39% of all carbon dioxide emissions in 2016 [19]. In Auckland (NZ’s largest city), congestion costs range from NZD $0.25 to $1.25 billion a year, equivalent to USD $0.16 to $0.80 billion annually [20]. The importance of mode shift from private vehicle travel, particularly in urban areas, has been acknowledged in the latest Government Policy Statement on Land Transport [21] and recent policy recommendations [22,23].

Travelling to school by car diminishes opportunities for physical activity in young people [24] and contributes to traffic congestion at peak times as well as associated noise and air pollution [25]. NZ adolescents who usually travelled to school actively accumulated approximately 15 min more moderate-to-vigorous physical activity during the week than those travelling by vehicle [26]. Reliance on private vehicles may also have long-term consequences, with childhood and adolescence transport habits continuing into adulthood [27,28,29]. Urban adolescents in NZ prefer car-based transport and the majority intend to learn to drive [30]. Encouraging active transport to school (and reducing reliance on private vehicles) is a recommended priority for reducing the rates of motorised transport and increasing rates of active transport in NZ [22,23]. 

A complex set of individual, family, social, built environment, and policy factors have effects on travel mode(s) to/from school. Increasing distance from home to school is the strongest predictor of motorised transport to school [31,32,33]. In the US, distance to school increased between 1969–2001, potentially accounting for half the decline in active travel during that period [31]. Higher rates of motorised transport to school have been observed among adolescents versus children [34,35] (at least partly due to increasing distance from home to secondary versus primary school) and girls versus boys [32] (although cross-country variations exist [36]). Social norms, family and peer support for active transport, trip-chaining practices, and adolescents’ and their parents’ concerns related to traffic and personal safety are also important [37,38,39,40,41,42,43]. Similarly, built environment features of home and school neighbourhoods (including neighbourhood walkability) may affect active transport to school [32,44,45]. Finally, policy factors, including school zoning, school choice policies, and public transport availability and/or subsidised school bus services, can have significant impacts on school car travel [46,47,48]. In NZ, the 1989 Education Act gave parents and students more school choices [49]; NZ adolescents enrolled in more distant schools travelled greater distances and had higher rates of motorised transport to school than those enrolled in the closest school [46]. 

With some exceptions [50,51,52], most previous studies compared active transport to school with “motorised transport” [32,39,53], combining trips by private vehicles, public transport, and/or subsidised transport such as school buses and taxis. Factors related to the use of each transport mode are likely different and should be examined separately. In primary school children, parents’ travel to work, parental attitudes, number of vehicles in the household, and distance to school were factors associated with car travel [50]. The availability, cost, convenience, and quality of public transport may affect the use of private vehicles for school travel in a particular geographic setting [51,54]. Public transport is usually accompanied by walking and/or cycling between the origin, the transit stop, and the destination [55], so public transport use can contribute to daily moderate-to-vigorous physical activity [56,57]. Social norms, support from peers and parents, parental perceptions of different transport modes, built environment features of the home and school neighbourhoods, and perceptions of traffic and personal safety may influence parents’ and young people’s preferences for travel mode to school [30,58]. 

This paper extends a previous meta-analysis of the built environment correlates of active transport to school in NZ children and adolescents conducted using individual-level data from five studies [32]. This article focuses on adolescents, given the lower levels of physical activity, lower rates of active transport to school, higher levels of independent mobility, and greater distance to school in this age group compared with younger children [31]. In addition, the associates of car-based travel to school may not necessarily produce opposite results from active transport to school. Given the dominance of private vehicle travel in NZ, understanding the correlates of private vehicle travel to school in a local context is essential to inform future interventions to reduce the rates of driving to/from school (in addition to the efforts to promote active transport to/from school). This meta-analysis examined sociodemographic characteristics and built environment associates of travelling to school by car compared with using active transport among NZ adolescents. 

## 2. Materials and Methods 

### 2.1. Study Context

NZ is an island nation in the South-Western Pacific Ocean with an estimated resident population of approximately five million people [59]; most residents live in urban areas. Tāmaki Makaurau (Auckland City) is NZ’s largest city, home to approximately one-third of the country’s population [60]. Te Whanganui-a-Tara (Wellington) is the second most populated city (11% of population) and Ōtepoti (Dunedin) is the seventh largest city in NZ (population: 120,000). The country has a very high human development index [61], although significant health inequities exist [62]. Urban and road design practices across the country have predominantly prioritised motorised travel (although policy changes to support increased active transport modes are occurring), underpinned by logics of “efficiency” and “speed optimisation”, shared by many countries worldwide. NZ’s transport system is dominated by private vehicle car ownership [63] and use [9]. Adolescents in NZ can begin the Graduated Driver Licencing System at 16 years [12], with a number of conditions on when, where, and with whom they can drive as they progress through the “learner”, “restricted”, and “full” licence stages. In 2011, NZ increased its driver age from 15 to 16, as a road-safety measure [64]. 

### 2.2. Identification of Primary Data Sources

NZ studies that had investigated objectively assessed neighbourhood built environment features in relation to child or youth travel mode to school were identified using a systematic literature search protocol as previously described [32] and outlined briefly below. 

#### 2.2.1. Sources and Search Strategy

The following databases were searched: ABI/Inform, Aotearoa New Zealand International Development Studies Network, Australian/New Zealand Reference Centre, New Zealand Educational Theses Database, NewzText (NZ media), NZResearch.org.nz (NZ theses), Scholarly Commons/Institutional Repository, Scopus, SPORTDiscus, and Web of Science. Search terms included: “active travel/transport”, “mode* travel/transport”, “school”, “Zealand”, “walk*”, “bik*”, “trip*”, “car*”. In addition, Government websites and Google were searched for relevant reports and literature. Searches were conducted between June and July 2016 and limited to English language literature published between January 1990 and June 2016. 

#### 2.2.2. Eligibility Criteria

The original search included empirical research (experimental, longitudinal, cross-sectional) that (1) included NZ children or youth aged between 5–19 years; (2) reported travel mode to school, and (3) reported geographic information systems (GIS)-derived measures of the home-neighbourhood built environment. Published and unpublished academic literature and grey literature (such as research reports) were all eligible. The meta-analysis reported here analysed data from adolescents only (age 13–19 years) and those that used either motorized or active transport to school.

#### 2.2.3. Study Identification

**Figure 1:**
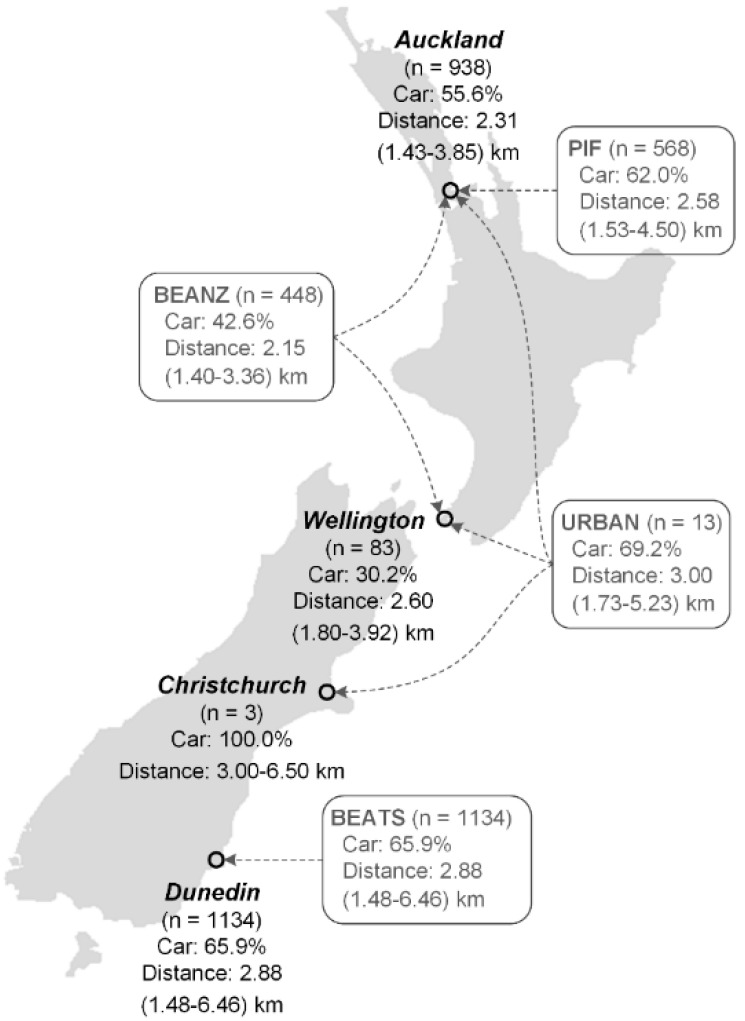
Locations, number of participants, and rates of travelling to school by car across four cities and four studies included in this meta-analysis. Distance is shown as median (25th–75th percentiles) except for the city of Christchurch where the range of distance (minimum–maximum) is displayed due to the small sample size (*n* = 3).

A flow chart for literature searching, screening, and inclusion derived from the Preferred Reporting Items for Systematic Reviews and Meta-Analyses (PRISMA) has been published previously [32]. Briefly, 23,956 records were screened, 49 full-text articles were assessed for eligibility, 12 full-text articles were selected for inclusion, and eight studies were identified as eligible for inclusion. Five out of eight study investigators agreed to provide data; these studies were included in the original individual-level participant meta-analysis [32]: BEANZ (Built Environment and Adolescent New Zealanders) [65], BEATS (Built Environment and Active Transport to School) [66], KITC (Kids in the City) [67], PIF (Pacific Islands Families) [68] and URBAN (Understanding the Relationship between Activity and Neighbourhoods) [69]. All studies had ethics approval from relevant institutions. This meta-analysis analysed data from adolescents only (age 13–19 years) and therefore included participant data from four studies: BEANZ, BEATS, PIF and URBAN (Figure 1). 

### 2.3. Data Preparation and Synthesis

Individual-level variables were collated as follows: participant demographics (age, sex, ethnicity, number of siblings, and number of individuals in a household), school characteristics (type of school attended and school decile (an area-level measure of socioeconomic status derived from households in a school’s neighbourhood [70])), school travel mode, and GIS-derived measures (home-to-school distance, residential density, intersection density, and land use mix) using a 400 m and 1 km street network buffer around each participant’s household address. Information about individual study methods were also collected. 

#### 2.3.1. Participant Characteristics and Mode of Travel to School

Age was classified into five groups (“13”, “14”, “15”, “16”, and “≥17” years) to detect any possible nonlinearity in relationships and to ensure sufficient number of participants at each age. Imputation was used for 19 participants with missing age data, using the mean age for participants from the same school year. Participants were categorised into five ethnic groups (“Māori”, “Pacific”, “Asian”, “Other”, and “NZ European”) based on NZ’s prioritised ethnicity categories [71]. Prioritised ethnicity involves categorising participants who have reported multiple ethnicities into one ethnic group, following the prioritised order listed above. The NZ index of deprivation 2013 is an area-level measure of deprivation (ranging from 1 = least deprived to 10 = most deprived), generated using nine measures of deprivation from Census data (i.e., income, home ownership, employment status, qualifications, family structure, housing, access to transport, and communications) [72]. New Zealand Deprivation index decile data were recoded into tertiles (1–3 = low deprivation; 4–7 = medium deprivation; 8–10 = high deprivation). Using body mass index (calculated from measured height and weight data [kg/m^2^]), participants were categorised as “underweight”, “normal weight”, “overweight”, or “obese” using the World Health Organization’s growth curve data [73]. Data on household size and number of siblings were also collected. In the first instance, frequency distributions of categorical data were checked and re-categorised if necessary (i.e., age, deprivation, household size, siblings) if a disproportionately low number of responses was observed in one or more categories. 

Due to differences in collection of school travel data across the four studies (details are available elsewhere [32]), travel mode data were initially dichotomised as passive (i.e., car, bus, motorbike) and active modes of travel (i.e., walk, bicycle, skateboard, and scooter) on ≥3 days per week or “most/all the time” [32]. For the purpose of this analysis, adolescents travelling to school by public transport or school bus (*n* = 614) as well as those using mixed modes to travel to school (*n* = 199) were excluded from the passive travel modes. The dichotomised school travel variable of “car” versus “active transport” was used in this analysis. 

#### 2.3.2. School Level Deprivation

Ministry of Education data [74] were used to identify school decile values as a measure of school level deprivation. School decile is based on a range of socio-economic indicators calculated for the areas that a school’s student population resides in. Decile 1 includes the 10% of schools with the highest proportion of students from low socioeconomic communities whereas decile 10 includes the lowest proportion of those students. In this study, school deciles were recoded into three groups (deciles 1–3 = “high deprivation”; deciles 4–7 = “medium deprivation” and deciles 8–10 = “low deprivation” schools).

#### 2.3.3. Built Environment Variables

Built environment variables included distance from home to school and home neighbourhood dwelling density, land use mix, and intersection density, as described previously [32]. Briefly, the distance of the shortest path from home to school was calculated using street network data that contained pedestrian paths in ArcGIS 10.2 (ESRI, Redlands, CA, USA). Dwelling density was calculated as an area weighted average number of private occupied dwellings using the 2013 Census data [75]. Land use mix (residential, commercial, public open space, industrial, recreation, or other) was calculated using parcel-level zoning data from territorial authority (sourced in 2013/2014) and Zenbu online business directory (extracted in 2014). Intersection density was calculated as the number of three-way or more intersections per square kilometre using road centreline data from Land Information New Zealand (www.linz.govt.nz). Built environment variables (dwelling density, intersection density, and land use mix) were organised into quartiles.

### 2.4. Statistical Analyses

A one-step individual participant data (IPD) meta-analysis approach was taken. The more traditional meta-analyses utilize aggregate level data (e.g., effect size estimates), which are extracted from the individual studies and synthesized into an overall estimate of effect size. In a one-step approach, the original IPD from all studies are modelled simultaneously. This allows for consistent approaches to calculation of explanatory factors and outcomes, as well as consistent handing of confounding factors and design effects. All analyses were performed in Statistical Analysis System (SAS) statistical package (v9.4, SAS Institute, Cary, NC, USA).

The final model was developed utilizing a stepwise logistic regression process. An initial bivariate logistic regression variable selection step was performed to determine what variables were suitable candidates for inclusion in the final model. A criterion of *p* < 0.20 was set for inclusion in the model, with age, sex, and ethnicity included as fixed effects, being known correlates of active transport versus transport by car to school behaviour [53,76,77,78]. The study effects were managed by stratifying by study. Individual study results for the full model were also calculated and presented using forest plots.

## 3. Results

Overall, 2163 participants from four NZ studies [65,66,68,69] were included in this meta-analysis: 51.2% were female and 54.5% of NZ European ethnicity (Table 1). Overall, 71.1% were 13–15 years of age (secondary school students under driving licence age), and 28.9% were 16 years of age or older (eligible to start the learning-to-drive process). On average, participants lived in households of 4.5 (±SD = 1.1) individuals and had 1.8 (±SD = 1.0) siblings. Median distance to school was 2.6 km (interquartile range: 1.5–5.0 km). Ten percent of the participants travelled >11 km to school; 5% travelled >16 km and 1% travelled >28 km (maximum distance: 81 km). In this sample, 60.1% of participants travelled to school by car, and 39.9% used active transport (Table 1). 

Results of the bivariate analyses for odds of travelling to school by car are presented in Table 1. School decile, NZ deprivation score, household size, home-to-school distance, and built environment features of the home neighbourhood (dwelling density, intersection density, and land use mix; 400 m and 1 km buffers) were related to travelling to school by car at *p* < 0.10 and were taken forward into the multiple variable modelling, along with the fixed effects (age, sex, ethnicity, and number of siblings).

**Figure 2:**
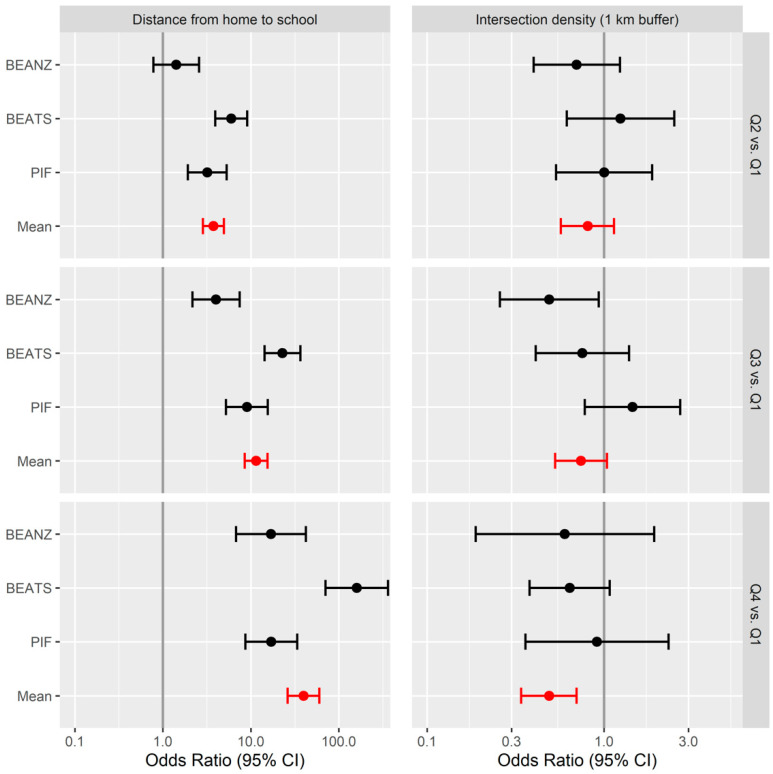
Forest plot comparing quartile 1 with quartiles 2, 3, and 4 for distance to school and home neighbourhood intersection density (1 km buffer) associations with car travel to school. BEANZ = Built Environment and Adolescent New Zealanders Study; BEATS = Built Environment and Active Transport to School Study; PIF = Pacific Islands Families Study; Q = quartile. Note: Due to small sample size, data from URBAN Study are not presented here.

The final multivariable model with results of the logistic regression analysis is presented in Table 2 (see also Figure S1 in online supplement). After removal of non-significant variables at *p* > 0.05, age, school decile, distance to school and intersection density of the home neighbourhood (1 km buffer) were significantly related to car travel to school. Sex and ethnicity were retained in the model as fixed factors but were not significantly associated with car travel to school. Increasing distance to school was the most statistically significant associate of car transport to school (*p* = 0.02). Compared with their peers living less than 1.5 km from school, adolescents living 1.5–2.5 km from their school had nearly four times higher odds and those living 2.6–4.9 km from their school had 11.5 times higher odds of car transport to school. The odds of car travel to school increased nearly 40 times among those living 5.0 km or more from their school compared with their peers living less than 1.5 km distance from school. An inverted U-shape relationship was observed between the school decile and rates of car transport to school. Adolescents enrolled in medium deprivation schools had higher likelihood of relying on car transport to school compared with those attending high deprivation schools whereas the odds of car travel to school were not significantly different between low and high deprivation schools. School decile (school deprivation) and neighbourhood deprivation index were highly but negatively correlated (Spearman correlation = −0.62), and school decile was the more significant associate for this analysis. The odds of car transport to school were halved among adolescents living in neighbourhoods with the highest quartile of intersection density compared with those living in neighbourhoods with the lowest quartile of intersection density. Land mix use and dwelling density were not significant correlates of travelling to school by car in the final model. A forest plot depicting the odds of travelling to school by car across intersection density and distance to school quartiles is shown in Figure 2. For each distance quartile, the odds of travelling by car were highest in the BEATS Study.

## 4. Discussion

### 4.1. Sociodemographic and Built Environment Associates of Car Travel to School

This meta-analysis examined the associates of car travel to school among adolescents in NZ. The key findings are that in addition to greater distance to school, both school level deprivation and low intersection density in home neighbourhoods are associated with higher likelihood of NZ adolescents travelling to school by car compared with active transport. 

Multiple studies conducted both internationally [31,33] and in NZ [32,41,53] have found that greater home-to-school distance is the strongest predictor of adolescents’ reliance on motorised transport (including both private vehicles and public transport) for school journeys. Unsurprisingly, this study also shows that home-to-school distance is the strongest predictor of car travel to school among adolescents living in a country with car-dominated transport system. Recent BEATS Study findings from the city of Dunedin estimated that 11.5% of city-wide car trips (95% CI 7.8–16.8%) and 12.5% of car distance driven (95% CI 8.8–17.7%) were related to secondary-school travel [42]. The same study reported that modest reductions in private vehicle traffic, particularly around the schools, would be observed if adolescents enrolled in their closest school [42], which would inevitably result in reduced distance for school travel [46]. 

Given the importance of distance to school, future urban planning efforts and construction of new schools should take into account high school location with respect to proximity and access to residential areas, including considering reasonable distances for adolescents’ walking and cycling to school in a local context: such distances vary across countries (ranging from 1.4–3.0 km for walking [45,79,80,81] and 3.0–8.0 km for cycling to school [80,81,82,83]). The findings of this study also show notable variations in the effects of distance from home to school on the car travel to school across studies conducted in different NZ cities. The variations in reasonable walking/cycling distances are influenced by social norms [40], peer and parental perceptions of and support for active transport to school [37,58], and social capital [84,85]. Topography [37], climate [86], built environment features (discussed further below), and bodily capabilities [87] also play important roles in understanding distances adolescents may be able and willing to walk or cycle to school. While policy can learn from international counterparts, spatially differentiated and geographically sensitive responses are important, particularly in accounting for the different contexts between countries, regions, cultures, and bodies. 

Future urban planning initiatives should also consider providing alternative transport options, such as high-quality public transport services, for adolescents who live beyond reasonable walking and cycling distances to school. Although public transport (including school bus) users and travellers using mixed modes were excluded from the current meta-analysis, it is important to note that over a quarter of the total sample used these travel modes (20.6% public transport, 6.7% mixed modes). Recent findings showed that major barriers for adolescents’ use of public transport to school in Dunedin, NZ, related to public bus services (cost, trip duration, and infrequent services), infrastructure (e.g., lack of bus shelters), and information (no real-time information) as well as social factors (chauffeuring children, and convenience of trip chaining activities), built environment features (distance to school or to bus stops), and natural environment (hilly terrain, the weather) [54]. 

The results of this study show that adolescents reduce their reliance on cars as they get older from 13 to 15 years of age, but the likelihood of using a private car to travel to school increases again from 16 years of age, which coincides with the minimum age for gaining a driver’s licence in NZ [12]. This suggests there may be a short period in early adolescence where independence is sought from active transport modes (i.e., walking or cycling), rather than relying on family members for motorised transport. However, from the age where licensing is possible, this independence is then replicated in learn-to-drive behaviours. Research has shown that gaining a driver’s licence has a range of meanings which go beyond the skillset and legal capacity to drive a motor vehicle [88]. These include a rite-of-passage into adulthood; perceptions of freedom, autonomy, and independence (from family members and bus timetables); and requirements by employers. This may suggest that finding ways to continue the association of active modes with independence through adolescence could increase active transport to school and reduce motorised mobility. Such interventions to delay or prevent adolescents learning to drive could have additional benefits including reduced injury and fatality in collisions [89].

In addition to distance to school, we found school decile (school deprivation) had an inverted U-shaped relationship with the rates of travelling to school by private vehicles. Adolescents enrolled in medium deprivation schools had lower likelihood of relying on car transport to school compared with those attending high deprivation and low deprivation schools. Previous studies found a greater reliance on car travel to school among youth residing in households with higher household socioeconomic status compared with their peers from less affluent families [34,90]. The finding of the present study should be interpreted in light of high rates of private vehicle ownership [8] and use [9] in NZ coupled with low rates of public transport to school [54], partly due to limited provision and use of public transport in many NZ cities [9] and an education system that supports choosing schools based on social factors, facilities/programmes, and school type rather than proximity to home [47]. Future studies should examine to what extent the higher likelihood of car transport to medium deprivation NZ schools observed in this study is related to school choice [46], and the impacts of school environment, such as school-specific social norms, which encourage or hinder car transport to school [91]. Moreover, the relationship between parent/caregiver employment and transport to school mode needs to be better understood, as working hours and work location may differ between parents in higher and lower deprivation schools compared with medium deprivation schools. 

Finally, in the present study, NZ adolescents residing in urban neighbourhoods with low intersection density (often observed in sprawling neighbourhoods) had a higher likelihood of travelling to school by car, even after accounting for distance to school. There is a paucity of literature that has examined motorised/car travel as the dependent variable, with most evidence exploring active modes as the outcome. Some previous studies found that high intersection density was associated with high rates of active transport to school [92], but contrasting findings were also reported in some countries [93,94,95], with higher intersection density being associated with lower rates of active transport to school. The inconsistent findings of the objectively measured intersection density may be due to the heterogeneity in GIS methods including buffer areas (e.g., home, school, and route) and buffer calculation (e.g., distance and network) [96]. Nevertheless, there is promising evidence that built environment features such as walkability (which is often measured using intersection density/street connectivity, land use mix, and dwelling/population density) are positively associated with physical activity and active travel behaviour [97]. Although public transport users were excluded from this study, low intersection density (particularly in association with urban sprawl) is often associated with low provision of public transport, which may explain some other studies’ findings regarding active travel.

### 4.2. Implications of Study Findings

To reduce the rates of car transport to school and the associated negative effects on adolescents’ health and the environment, disincentivising private vehicle use may be as important as incentivising active modes. Perceived convenience of being driven to school (including trip chaining) [92,98] is a barrier to active transport to school, particularly in car-dominated societies. NZ adolescents have ongoing preferences for car-based transport [30], and more than half of adolescents living within cycling distance to school found being driven to school convenient [37]. Initiatives to disincentivise car travel to school could include raising parking prices around schools and parents’ workplaces to discourage parents and adolescents from driving to/from school. In addition, initiatives to incentivise other transport modes could include improving bus infrastructure and services, providing public transport subsidies, and changing perceptions of public transport use and users [54]. Future initiatives to reduce car transport to school should also consider creating safe walking and cycling routes to school with drop-off and pick-up points within walking and cycling distance [99] to encourage use of mixed transport modes for school travel even among those adolescents who live beyond walking and/or cycling distance to school. 

### 4.3. Study Strengths and Limitations

The strengths of this study include the large sample size of participants from three major urban centres in NZ and analysis of participant-level data. Limitations include analysis of data from cross-sectional studies, absence of data on travel from school, reliance on data collected only in urban settings, and lack of analysis of correlates of car travel to school in younger children. Future studies should examine what adolescents and parents consider to be acceptable commute times and distances for walking, cycling, and public transport to school and use that information to tailor local initiatives to reduce the rates of private vehicle travel to school.

## 5. Conclusions

The results of this meta-analysis suggest that in addition to greater distance to school, low and high school-level deprivation and built environment characteristics (low intersection density in the home neighbourhood) are associated with higher likelihood of car travel to school compared with active transport among NZ adolescents. Interventions aimed to reduce private vehicle travel to/from school in NZ should be tailored to the school context and address built environment factors with the main focus on reducing the negative impact of distance to school through encouraging use of public transport and mixed modes. Beyond NZ, these findings show the importance of geographical specificities in designing interventions, which account for the socio-cultural, built environment, and institutional details of the towns, cities, and school environment.

## Figures and Tables

**Table 1:** Bivariate correlates of travelling to school by car.

	Total Sample	Car Transport *n* (%)	Active Transport *n* (%)	OR ^1^ (95% CI ^2^)	*p*-Value
Age (years)					
13	197	124 (62.9)	73 (37.1)	0.80 (0.55, 1.16)	
14	848	512 (60.4)	336 (39.6)	0.71 (0.53, 0.95)	
15	491	280 (57.0)	211 (43.0)	0.64 (0.47, 0.86)	
16	277	151 (54.5)	126 (45.5)	0.60 (0.43, 0.84)	
17 or older (Reference)	349	232 (66.5)	117 (33.5)		0.02
Sex					
Male (Reference)	1056	611 (57.9)	445 (42.1)		
Female	1107	688 (62.1)	419 (37.9)	1.12 (0.94, 1.34)	0.20
Ethnicity					
New Zealand European (Reference)	1176	719 (61.1)	457 (38.9)		
Māori	145	76 (52.4)	69 (47.6)	0.66 (0.46, 0.94)	
Pacific	567	360 (63.5)	207 (36.5)	1.31 (0.86, 1.99)	
Asian	105	55 (52.4)	50 (47.6)	0.98 (0.65, 1.50)	
Other	166	87 (52.4)	79 (47.6)	0.74 (0.53, 1.04)	0.03
Weight status category					
Underweight	14	10 (71.4)	4 (28.6)	1.99 (0.58, 6.77)	
Normal weight (Reference)	1174	682 (58.1)	492 (41.9)		
Overweight	455	283 (62.2)	172 (37.8)	1.14 (0.90, 1.43)	
Obese	428	264 (61.7)	164 (38.3)	1.10 (0.84, 1.44)	0.50
New Zealand Deprivation score					
Low deprivation (Reference)	726	488 (67.2)	238 (32.8)		
Medium deprivation	677	365 (53.9)	312 (46.1)	0.55 (0.44, 0.69)	
High deprivation	739	430 (58.2)	309 (41.8)	0.46 (0.35, 0.61)	<0.0001
Number of individuals living in a household					
2 (Reference)	72	37 (51.4)	35 (48.6)		
3	345	185 (53.6)	160 (46.4)	1.01 (0.60, 1.70)	
4	676	422 (62.4)	254 (37.6)	1.55 (0.94, 2.55)	
5	483	294 (60.9)	189 (39.1)	1.42 (0.85, 2.36)	
6 or more	578	355 (61.4)	223 (38.6)	1.51 (0.90, 2.53)	0.02
Number of siblings					
0 (Reference)	214	108 (50.5)	106 (49.5)		
1	693	428 (61.8)	265 (38.2)	1.33 (0.97, 1.84)	
2	555	332 (59.8)	223 (40.2)	1.14 (0.82, 1.58)	
3 or more	701	431 (61.5)	270 (38.5)	1.11 (0.80, 1.54)	0.22
School decile					
Low deprivation	950	625 (65.8)	325 (34.2)	2.00 (1.60, 2.42)	
Medium deprivation (Reference)	716	369 (51.5)	347 (48.5)		
High deprivation	494	302 (61.1)	192 (38.9)	1.30 (0.87, 1.94)	<0.0001
Distance from home to school					
Q1 < 1.48 km (Reference)	539	121 (22.4)	418 (77.6)		
Q2 1.48–2.60 km	540	264 (48.9)	276 (51.1)	3.62 (2.76, 4.73)	
Q3 2.61–4.99 km	539	412 (76.4)	127 (23.6)	11.86 (8.88, 15.83)	
Q4 ≥ 5.00 km	540	501 (92.8)	39 (7.2)	42.97 (29.23, 63.19)	<0.0001
Home neighbourhood dwellings density (per km^2^)					
[400 m buffer]					
Q1 < 743 (Reference)	539	419 (77.7)	120 (22.3)		
Q2 743–970	540	333 (61.7)	207 (38.3)	0.47 (0.36, 0.62)	
Q3 971–1139	540	291 (53.9)	249 (46.1)	0.35 (0.27, 0.46)	
Q4 ≥ 1140	540	255 (47.2)	285 (52.8)	0.28 (0.21, 0.36)	<0.0001
[1 km buffer]					
Q1 < 643 (Reference)	539	437 (81.1)	102 (18.9)		
Q2 643–903	540	326 (60.4)	214 (39.6)	0.38 (0.28, 0.50)	
Q3 904–1028	540	291 (53.9)	249 (46.1)	0.29 (0.22, 0.39)	
Q4 ≥ 1029	540	244 (45.2)	296 (54.8)	0.22 (0.16, 0.29)	<0.0001
Home neighbourhood intersection density (per km^2^)					
[400 m buffer]					
Q1 < 26.7 (Reference)	540	390 (72.2)	150 (27.8)		
Q2 26.7–39.0	539	329 (61.0)	210 (39.0)	0.64 (0.49, 0.84)	
Q3 39.1–52.4	543	313 (57.6)	230 (42.4)	0.52 (0.40, 0.68)	
Q4 ≥ 52.5	541	267 (49.4)	274 (50.6)	0.30 (0.23, 0.40)	<0.0001
[1 km buffer]					
Q1 < 26.2 (Reference)	539	401 (74.4)	138 (25.6)		
Q2 26.2–35.2	540	329 (60.9)	211 (39.1)	0.65 (0.49, 0.86)	
Q3 35.3–45.4	543	312 (57.5)	231 (42.5)	0.48 (0.36, 0.63)	
Q4 ≥ 45.5	541	257 (47.5)	284 (52.5)	0.20 (0.15, 0.27)	<0.0001
Home neighbourhood land use mix					
[400 m buffer]					
Q1 < 0.01 (Reference)	545	336 (61.7)	209 (38.3)		
Q2 0.01–0.19	502	293 (58.4)	209 (41.6)	1.25 (0.95, 1.64)	
Q3 0.20–0.31	519	315 (60.7)	204 (39.3)	1.63 (1.22, 2.19)	
Q4 ≥ 0.32	578	342 (59.2)	236 (40.8)	1.51 (1.13, 2.03)	0.008
[1 km buffer]					
Q1 < 0.17 (Reference)	522	319 (61.1)	203 (38.9)		
Q2 0.17–0.31	519	300 (57.8)	219 (42.2)	1.32 (1.00, 1.73)	
Q3 0.32–0.40	520	321 (61.7)	199 (38.3)	1.52 (1.14, 2.03)	
Q4 ≥ 0.41	588	348 (59.2)	240 (40.8)	1.49 (1.12, 1.99)	0.02

^1^ OR = odds ratio; ^2^ CI = confidence interval; ^3^ Q = quartile.

**Table 2:** Final multivariate model of correlates of travelling to school by car.

	OR ^1^	95% CI ^2^	*p*-Value
Age (years)			
13	0.73	(0.46, 1.16)	
14	0.71	(0.50, 1.01)	
15	0.55	(0.38, 0.79)	
16	0.61	(0.41, 0.91)	
17 or older (Reference)			0.02
Sex			
Male (Reference)			
Female	1.24	(1.00, 1.53)	0.05
Ethnicity *			
New Zealand European (Reference)			
Māori	0.73	(0.47, 1.15)	
Pacific	1.59	(0.97, 2.59)	
Asian	1.01	(0.61, 1.68)	
Other	0.85	(0.57, 1.28)	0.12
School decile			
Low deprivation	1.95	(1.50, 2.53)	
Medium deprivation (Ref)			
High deprivation	2.85	(1.73, 4.72)	<0.0001
Distance from home to school			
Q1 ^3^ < 1.48 km (Reference)			
Q2 1.48–2.60 km	3.76	(2.86, 4.96)	
Q3 2.61–4.99 km	11.51	(8.53, 15.53)	
Q4 ≥ 5.00 km	39.71	(26.21, 60.15)	<0.0001
Home neighbourhood intersection density (per km^2^) [1 km buffer]			
Q1 < 26.2 (Reference)			
Q2 26.2–35.2	0.81	(0.57, 1.14)	
Q3 35.3–45.4	0.74	(0.53, 1.04)	
Q4 ≥ 45.5	0.49	(0.34, 0.70)	0.001

^1^ OR = odds ratio; ^2^ CI = confidence interval; ^3^ Q = quartile. * Note that ethnicity results are not significant but included as at least one study has a different ethnic mix (PIF Study) and as ethnicity is often a factor in similar studies.

## Supplementary Materials

The following are available online at https://www.mdpi.com/1660-4601/17/23/9138/s1. Figure S1: Correlates of travelling to school by car in the final multivariate model.
