# Supplementary material for: Sociodemographic and Built Environment Associates of Travel to School by Car among New Zealand Adolescents: Meta-Analysis
Source: Int J Environ Res Public Health. 2020 Dec 7;17(23):9138. doi: 10.3390/ijerph17239138 (PMC7730892; doi:10.3390/ijerph17239138)
Supplement: Supplementary file 1 [file ijerph-17-09138-s001.pdf]

International Journal of
Environmental Research and Public Health

MDPI

Online supplement

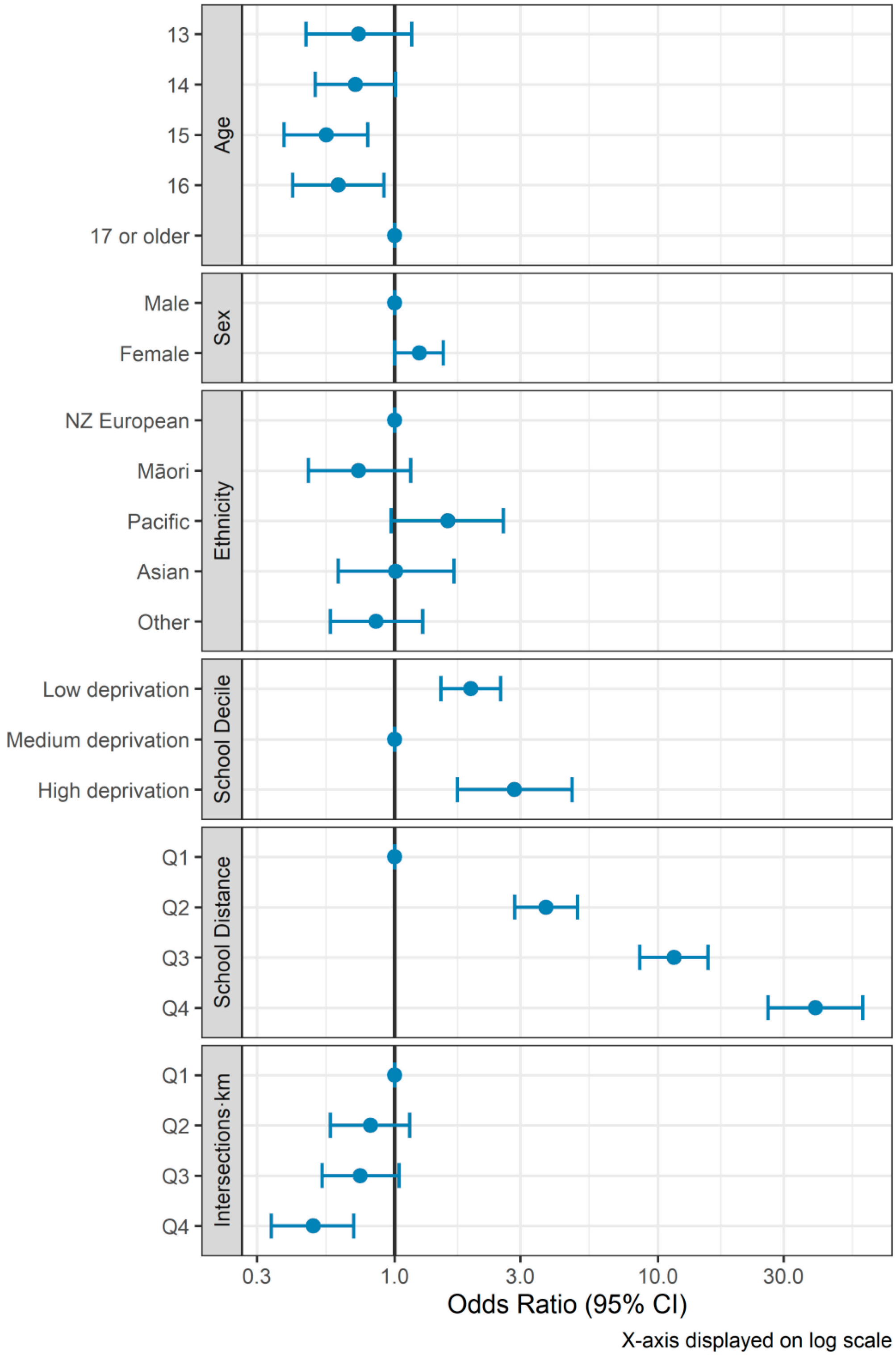

Figure S1. Correlates of travelling to school by car in the final multivariate model.
